# Craniotomies following acute traumatic brain injury in Finland—a national study between 1997 and 2018

**DOI:** 10.1007/s00701-022-05140-x

**Published:** 2022-02-04

**Authors:** Nea Nevalainen, Teemu M. Luoto, Grant L. Iverson, Ville M. Mattila, Tuomas T. Huttunen

**Affiliations:** 1 Faculty of Medicine and Health Technology, Tampere University, Tampere, Finland; 2 Department of Neurosurgery, Tampere University Hospital and Tampere University, Tampere, Finland; 3 Department of Physical Medicine and Rehabilitation, Harvard Medical School, Boston, USA; 4 Spaulding Rehabilitation Hospital and Spaulding Research Institute, Boston, USA; 5 Home Base, A Red Sox Foundation and Massachusetts General Hospital Program, Charlestown, MA USA; 6 Division of Orthopaedics and Traumatology, Department of Trauma, Musculoskeletal Surgery and Rehabilitation, Tampere University Hospital, Tampere, Finland; 7 Coxa Joint Replacement Hospital, Tampere, Finland; 8 Department of Cardio-Thoracic Surgery, Tampere Heart Hospital, Tampere University Hospital, Tampere, Finland

**Keywords:** Traumatic brain injury, Neurosurgery, Craniotomy, Epidemiology

## Abstract

**Background:**

A number of patients who sustain a traumatic brain injury (TBI) require surgical intervention due to acute intracranial bleeding. The aim of this retrospective study was to assess the national trends of acute craniotomies following TBI in the Finnish adult population.

**Methods:**

The data were collected retrospectively from the Finnish Care Register for Health Care (1997–2018). The study cohort covered all first-time registered craniotomies following TBI in patients aged 18 years or older. A total of 7627 patients (median age = 59 years, men = 72%) were identified.

**Results:**

The total annual incidence of acute trauma craniotomies decreased by 33%, from 8.6/100,000 in 1997 to 5.7/100,000 in 2018. The decrease was seen in both genders and all age groups, as well as all operation subgroups (subdural hematoma, SDH; epidural hematoma, EDH; intracerebral hematoma, ICH). The greatest incidence rate of 15.4/100,000 was found in patients 70 years or older requiring an acute trauma craniotomy. The majority of surgeries were due to an acute SDH and the patients were more often men. The difference between genders decreased with age (18–39 years = 84% men, 40–69 = 78% men, 70 + years = 55% men). The median age of the patients increased from 58 to 65 years during the 22-year study period.

**Conclusions:**

The number of trauma craniotomies is gradually decreasing; nonetheless, the incidence of TBI-related craniotomies remains high among geriatric patients. Further studies are needed to determine the indications and derive evidence-based guidelines for the neurosurgical care of older adults with TBIs to meet the challenges of the growing elderly population.

## Introduction

Acute intracranial bleeding can be life-threatening following traumatic brain injury (TBI). A foundation of the emergency management of TBI is to identify the presence of intracranial bleeding and determine whether neurosurgery is required to evacuate blood from the cranial vault. Intracranial abnormalities, visible on day-of-injury computed tomography (CT), are relatively common and occur in a substantial proportion of people presenting to the emergency department (ED) following acute head trauma [49]. The rate of abnormalities increases in association with the severity of injury, as measured by the Glasgow Coma Scale (GCS), whereby those with a GCS score of 15 have fairly low rates [13, 17] but those with GCS scores of 14, 13, and lower have much higher rates [15]. The large majority of those cases, however, do not require neurosurgery [12]. The rates of people requiring neurosurgery for an intracranial abnormality, in consecutive series of patients presenting to the ED following head trauma, vary across studies, but are often reported to range between approximately 5% and 37% in the group of moderate to severe TBI [9, 21, 25, 33].

People who sustain severe TBIs are at risk for lifelong disability [24]. In Europe, TBI is responsible for the greatest number of total years lived with disability resulting from trauma [24] and it is also one of the injuries with the highest hospital cost per capita [39]. A cost-analysis study in 2018 stated that in Finland the 1-year healthcare cost of all patients with TBI treated in an intensive care unit (ICU) was 122 million USD, and the total costs have increased over the past decade in part due to greater numbers of older adults being admitted to the ICU following injury [41]. As the world’s population increases and ages, emergency medicine and neurosurgery will need to adapt and develop the management of TBI in older adults. Increasing frailty and medical co-morbidities place elderly at risk of neurosurgical emergency after injury. Moreover, the use of antithrombotics in the elderly population puts people at risk for intracranial bleeding [1, 32].

There are no nationwide studies reporting the incidence of acute neurosurgical management of TBI in the adult population. Better understanding of the incidence rates and temporal trends of trauma-related craniotomies might modify clinical practice and aid health policy-making by informing preventive measures and healthcare resource utilization. The aim of this retrospective cohort study is to assess the national trends of craniotomies following acute TBI in the whole Finnish adult population in 1997–2018. We analyze incidence rates for acute craniotomies following acute subdural hematomas (SDH), epidural hematomas (EDH), and intracerebral hematomas (ICH). We hypothesized that the incidence of acute craniotomies, especially among the elderly, has increased during the last two decades.

## Methods and materials

Patient data was gathered retrospectively from the Finnish Care Register for Health Care (FCRHC). Patients aged 18 years or older with their date of admission between January 1, 1997 and December 31, 2018 were included. The FCRHC is a nationwide database that includes data on patients discharged from hospital care and contains variables such as service providing unit, age and sex of the patient, duration of hospital stay, all diagnoses (ICD-10, International Classification of Diseases-10 code), external causes and type of accident, and all procedures performed during the hospital stay. Surgical procedures are classified according to NCSP (Nordic Classification of Surgical Procedures) [35]. Both private and public health service providers are obligated to submit information to the FCRHC ensuring excellent national coverage, accuracy, and extent of the database [16, 29, 47].

The main outcome measure for this population-based study was the annual number and incidence (per 100,000 people per year, hereinafter presented as X/100,000) of hospitalized patients with TBI treated for the first time with an acute neurosurgical operation in Finland. To identify patients treated with acute TBI-related craniotomy, the criteria for selecting the patients were as follows: any of the diagnosis codes were trauma to the head and any of the procedures performed were either evacuation of EDH (NCSP code: AAD00), evacuation of acute SDH (AAD05), or evacuation of traumatic ICH (AAD15). A Finnish version of the NOMESCO (Nordic Medico-Statistical Committee) procedural coding was used [35].

Using the pseudonymized personal identification number, we were able to identify patients hospitalized more than once due to the same procedure and included only the first-time registered hospitalization among the same procedure type. The annual mean population according to age and sex was derived from Statistics Finland Open data pages [36].

Selected data were adjusted by age and sex in order to examine the trend in annual incidence rates of acute TBI-related neurosurgical operations performed from 1997 to 2018 and to compare incidence rates between subgroups. Incidence rates calculated with the patients gathered from the FCRHC represent the whole adult population (aged 18 years or older) in Finland, and thus statistical estimation methods such as p-values and 95% confidence intervals were not calculated. The data analyses were performed using IBM SPSS Statistics 26.0 (Armonk, NY, USA).

The research protocol for the present study was approved by the research committee of the National Institute of Health and Welfare (Dnro THL/1800/5.05.00/2019). Formal ethical approval or written informed consents were not required, because all the study data were collected retrospectively from national registries, and the data were analyzed in a pseudonymized fashion.

## Results

**Fig. 1:**
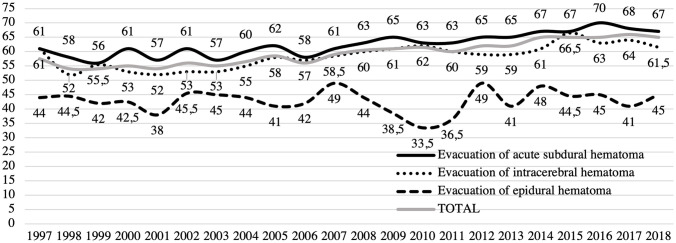
Median age of patients who underwent acute craniotomy following TBI between 1997 and 2018

**Fig. 2:**
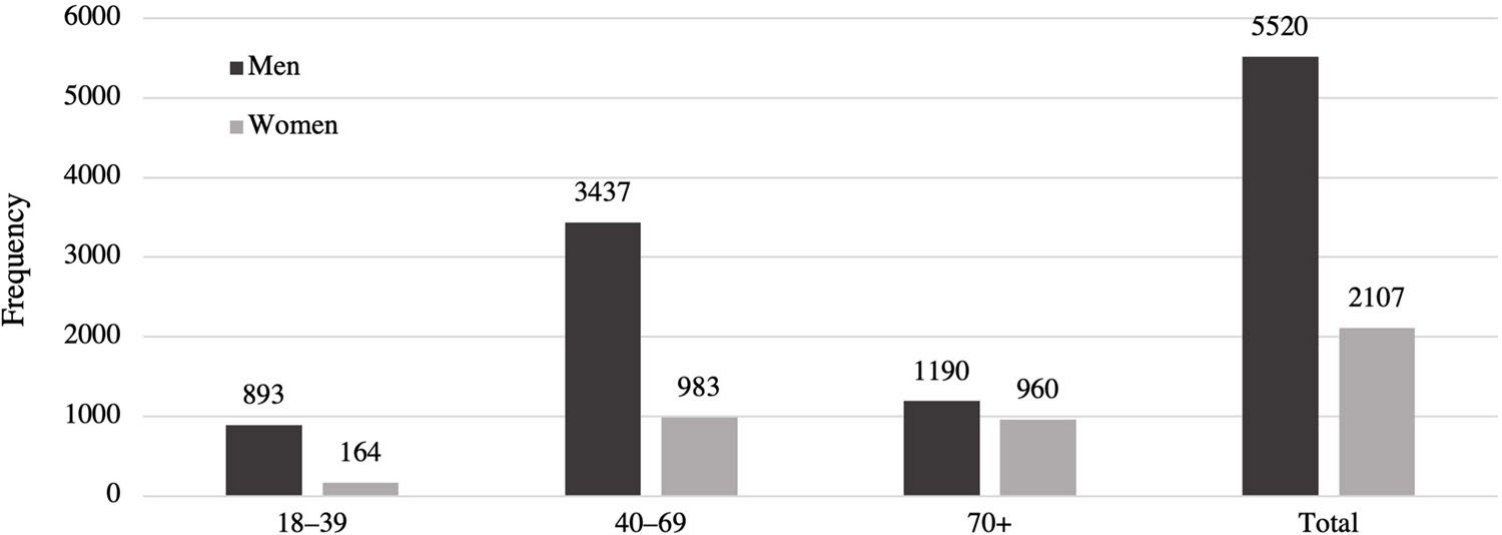
Number of patients undergoing neurosurgery between 1997 and 2018 by age and gender

A total of 7627 patients, 5520 (72%) men and 2107 (28%) women, underwent acute craniotomy due to TBI in Finland between 1997 and 2018. Of these, 5235 were for acute SDH evacuation surgeries, 1300 were for traumatic ICH evacuation surgeries, and 1092 underwent surgery for the evacuation of EDH. The median age of the patients was 59 years and during the 22-year study period the median age increased from 58 years in 1997 to 65 years in 2018. Patients with acute SDH were the oldest (median age 63 years) while patients that underwent EDH surgery were the youngest (median age 43 years). The median age of patients with traumatic ICH was 57 years. Men were younger than women in all procedure groups (Table 1). The median age in men increased from 53 years in 1997 to 62 years in 2018, and in women from 69 years in 1997 to 71 years in 2018. The trends in median age between 1997 and 2018 among patients who underwent surgery are presented in Fig. 1. The age distribution of the study population is shown in Fig. 2. Over the entire study period, men were over-represented (72.4%; Fig. 2). The gender difference varied by age (18–39 years = 84.5% men; 40–69 years = 77.8% men; and 70 + years = 55.4% men; Fig. 2).

**Table 1:** Total number of procedures and median age of surgically treated TBI patients

		*n*	%	Median age during study period	Median age 1997 (years)	Median age 2018 (years)
Subdural hematoma	Total	5235	–	63	61	67
	Men	3665	70	60	56	66
	Women	1570	30	70	71	71
Intracerebral hematoma	Total	1300	–	57	61	62
	Men	1007	77	56	54	59
	Women	293	23	63	66	72
Epidural hematoma	Total	1,092	–	43	44	45
	Men	848	78	41	43	41
	Women	244	22	52	55	60
Total	Total	7627	–	59	58	65
	Men	5520	72	57	53	62
	Women	2107	28	67	69	71

**Fig. 3:**
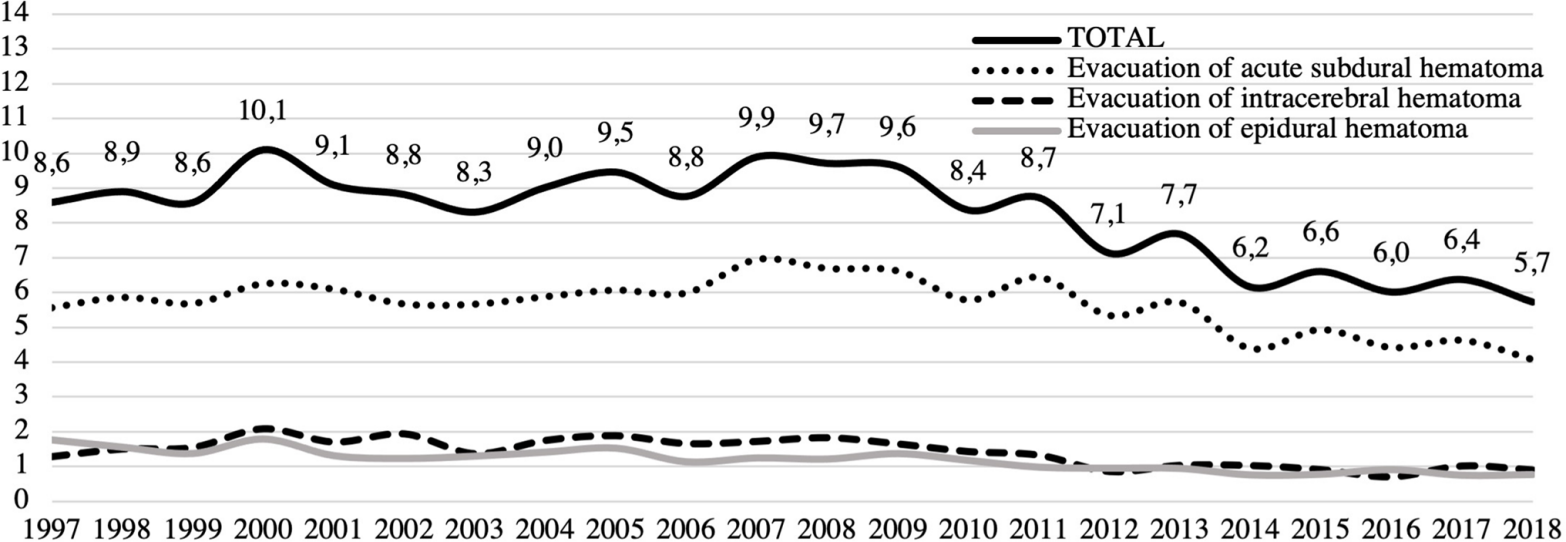
Incidence (per 100,000) of all acute craniotomies following TBI between 1997 and 2018

**Fig. 4:**
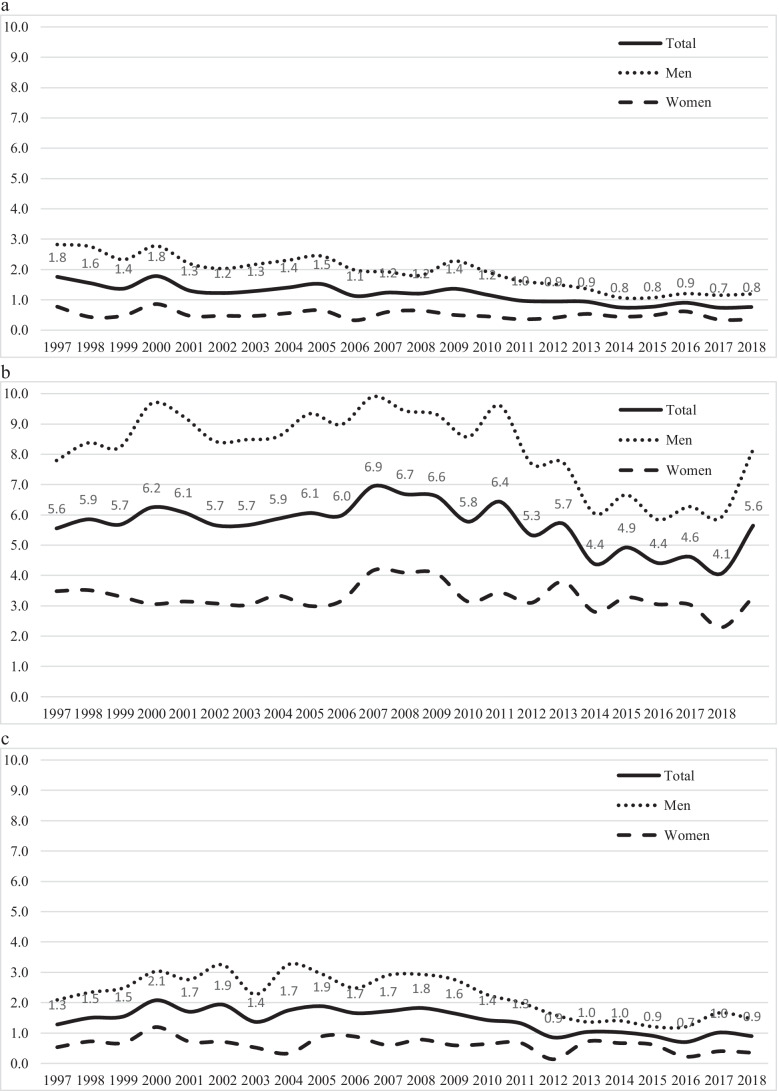
Incidence (per 100,000) of men and women who underwent EDH surgery (**a**), SDH surgery (**b**), and ICH surgery (**c**) between 1997 and 2018 in Finland

Through the study period, the total incidence of acute trauma craniotomies declined by 33% from 8.6/100,000 in 1997 to 5.7/100,000 in 2018 (Fig. 3). The incidence of acute SDH evacuation surgeries decreased from 5.6/100,000 in 1997 to 4.1/100,000 in 2018 (decrease of 27%). Corresponding numbers for traumatic ICH evacuation surgeries were 1.3/100,000 in 1997 and 0.9/100,000 in 2018 (decrease of 30%), and for EDH evacuation surgeries 1.8/100,000 in 1997 and 0.8/100,000 in 2018 (decrease of 57%). Men underwent surgery more frequently than women in all procedure groups (Fig. 4).

**Fig. 5:**
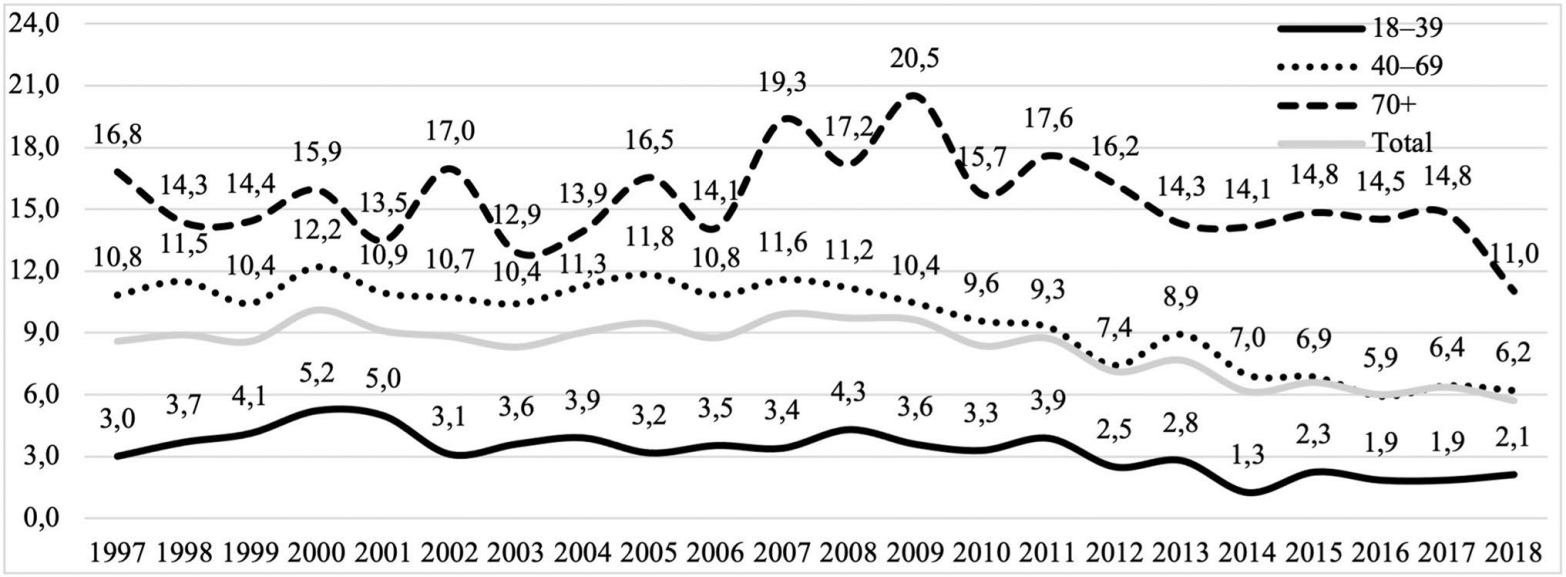
Incidence (per 100,000) of all acute craniotomies following TBI between 1997 and 2018 stratified by age groups

Evacuation of acute SDH was most common in the elderly population (aged 70 years and older) and least common among young adults (aged 18 to 39 years) between 1997 and 2018. In contrast, the evacuation of EDH was most common among young adults and least common among the elderly. Evacuation of traumatic ICH was as common among elderly as middle-aged patients (aged 40 to 69 years) while it was least common in the group of young adults (Table 2). In total, during the 22-year study period, neurosurgical operations following TBI were most common among patients aged 70 years and older with the incidence rate of 15.4/100,000. A decrease in the incidence of acute craniotomies following TBI was found in all age groups (Fig. 5).

**Table 2:** Incidence of procedures presented by sex and age group in the Finnish adult population in 1997–2018

Incidence/100,000 (*n*)	Age (years)		
	18–39	40–69	70 +
Subdural Hematoma	1.3 (434)	6.5 (3000)	12.9 (1801)
Men	2.1 (356)	10.1 (2317)	18.5 (992)
Women	0.5 (78)	2.9 (683)	9.4 (809)
Intracerebral hematoma	0.5 (151)	1.9 (888)	1.9 (261)
Men	0.8 (132)	3.1 (714)	3.0 (161)
Women	0.1 (19)	0.7 (174)	1.2 (100)
Epidural hematoma	1.4 (472)	1.2 (532)	0.6 (88)
Men	2.4 (405)	1.8 (406)	0.7 (37)
Women	0.4 (67)	0.5 (126)	0.6 (51)
Total	3.2 (1057)	9.6 (4420)	15.4 (2150)
Men	5.3 (893)	15.0 (3437)	22.2 (1190)
Women	1.0 (164)	4.2 (983)	11.1 (960)

## Discussion

We examined the nationwide trends of TBI-related acute neurosurgical operations in the Finnish adult population over a 22-year period. There were three main findings. First, during the study period, the total incidence rate of emergency craniotomies decreased, in total, by 33%. The decrease was seen in all procedure subgroups as well as in both men and women. Second, men required neurosurgery more frequently than women in all age groups. The male predominance decreased by age as the proportion of men was 85% between the ages of 18 and 39 years, 78% between 40 and 69 years, and 55% in those 70 years and older. Finally, the median age of patients increased during the study period (1997: 58 years; and 2018: 65 years).

As expected, acute SDH-related emergency craniotomies were the most common of the procedures and the median age of acute SDH patients was the highest of the three procedure groups (acute SDH, EDH, and ICH). The observed decrease in surgery rates was quite surprising because geriatric TBI in general has been considered to be a growing problem [19]. However, our findings were in line with Flynn-O’Brien and colleagues [10] who reported a decline from 36 to 7% in surgical intervention of severe TBI between 1995 and 2012. They also reported a likely multifactorial improvement in inpatient mortality in the study population. The contradiction between our findings and the previous reports of increasing geriatric TBI [19] could be explained in part by some reluctance to provide elderly patients with neurosurgery. There is a notable lack of guidelines and prognostic tools for geriatric TBI, thus complicating the neurosurgeon’s ability to make evidence-based decisions on surgical treatment. While present treatment guidelines are based on and targeted to younger patients, it is unclear which of the older patients actually benefit from emergency trauma neurosurgery [8, 14]. It is suggested that age alone should not serve as a contraindication for general neurosurgery [27, 50]. However, surgical decision-making based on local protocols and experience may exclude some elderly patients with TBIs who might benefit from neurosurgery [50]. It is also notable that the elderly patients often present with relatively high GCS scores, although suffering from a significant intracranial hemorrhage [46]. The initial clinical severity stratification of elderly TBI is often underestimated. The justification for performing a trauma craniotomy on a geriatric TBI patient is largely lacking evidence and might also be prone to implicit bias. For example, performing a trauma craniotomy on an 80-year-old patient with a GCS of 14 or 15 and an 11-mm-thick acute SDH is justified according to the current guidelines [4] derived from younger patient cohorts. However, many neurosurgeons would opt not to do surgery on such a case due to a poor long-term prognosis.

Overrepresentation of men was expected. Men are more prone to risk-taking behavior [5]. Men are reported to be predisposed to risk factors such as alcohol intoxication, as well as more likely to encounter situations such as violence and road traffic accidents leading to TBI [22, 28, 37, 44]. Our results align with previous reviews by Brazinova and colleagues [3] and Peeters and colleagues [38]. In both reviews, men are more likely to sustain TBIs than women. A considerable number of TBIs following road traffic accidents and violence incidents occur when the person is under the influence of alcohol [22, 28, 37, 44]. Posti and colleagues [40] reported that the overall incidence rate of fatal TBIs decreased by 4.1% annually between 2004 and 2016 in Finland. The decrease in TBI mortality was associated with the decrease in overall alcohol consumption. However, this temporal association was not evident in the older TBI population. Additionally, the incidence of road traffic accidents has decreased during our study period (statistics by Finnish Road Safety Council). It is possible that the decreasing rates of neurosurgery for EDH and ICH were related, in part, to societal alcohol consumption and decreasing rates of road traffic accidents. Unfortunately, we were unable to verify this using the present data. In high-income countries, the causes of TBI are gradually changing from high-energy injuries among younger patients to low-energy injuries, such as ground-level falls, among elderly. Older adults are at risk of fall-related TBIs due to frailty, comorbidity, and antithrombotic medication. In high-income countries, increasing life-expectancy predisposes older adults to TBI because older individuals stay mobile and live longer with chronic illnesses [19, 43]. To further prevent the increase of elderly falls and therefore TBI, prevention methods such as strength and balance training, use of vitamin D and calcium supplements, and professional home-hazard assessment is pointed to be effective [20].

Over the two–decade-long study period, the median age of patients who underwent acute TBI craniotomies increased by 7 years (from 58 to 65). The median age increased in all operation types. The most notable increase was found among male acute SDH patients whose median age increased by 10 years (from 56 to 66 years) during the study period. We assume the increase in the median age is due, in part, to the growing elderly population. Moreover, it can be speculated that in recent years neurosurgery is offered more frequently to elderly TBI patients compared to the end of the twentieth century. A growing body of evidence suggests that chronological age and TBI severity alone are insufficient to accurately predict outcome in older adults who sustain TBIs [6, 23, 26, 48]. Historically, elderly patients with mass lesions were considered to be cases with extremely poor outcomes regardless of treatment intensity. Thus, these patients with an assumed poor prognosis have been treated conservatively, simultaneously generating a self-fulfilling prophecy [31, 42]. Undoubtedly, some surgical treatment bias and nihilism toward elderly patients with TBIs still exists as pointed out earlier [45]. Moreover, the use of antithrombotic medication can contribute to the changing trends in the age distribution of trauma craniotomies [11]. The use of antithrombotics is increasing especially among individuals aged 65 years or older [1]. Approximately 30% of patients aged 65 years or older admitted to Level 1 trauma centers after a low-level fall and 33% of head trauma patients aged 55 years or older are on pre-injury antithrombotics [32, 34]. However, acute reversal strategies are available for some but not all oral anticoagulation drugs as well as antiplatelet agents [7, 51]. Prothrombin complex concentrate (PCC) therapy is shown to be effective in the rapid lowering of INR in the case of traumatic coagulopathy in patients with or without the pre-injury use of warfarin [2, 18]. The use of PCC therapy can quickly facilitate neurosurgical care of anticoagulated patients with ICH [2]. It is also shown that treatment with recombinant activated factor VII rapidly after the onset of ICH can reduce the growth of the hematoma [30]. It is uncertain whether the growth of the hematoma can be sufficiently reduced for conservative treatment to be used instead of surgical evacuation of the hematoma.

The strength of our study is the high quality of the FCRHC database for epidemiologic research. The data collection for FCRHC is mandatory for both public and private health care services and institutions in Finland and therefore has excellent nationwide coverage and accuracy [16, 29, 47]. Our strengths also include a long study period of 22 years and that the Finnish healthcare system is based on public healthcare entitled equally to everyone residing in Finland ensuring that the data reflects the actual annual need of emergency craniotomies.

The weakness of our study is that the FCRHC does not provide information about whether the patients hospitalized several times during the 22 years of follow-up had been admitted again due to the same TBI, revision of the previous operation, or due to a new TBI. Therefore, we included only the first hospitalization of each patient, hence every patient is represented only once in our data. Therefore, it is possible that we slightly underestimated the incidence of craniotomies following TBIs. Another weakness of the FCRHC is the lack of data about TBI severity, other treatments or outcomes, the patient’s comorbidities, or other risk factors such as medications or alcohol consumption that increase the risk of TBI. Furthermore, decompressive craniectomy procedures (code: AAK80) were excluded from the study due to NCSP coding inconsistencies. The AAK80 code was only introduced in 2007. During our study period, a total of 304 first-time registered decompressive craniectomies following TBI were reported in the database, with an annual incidence of 0.1–0.9 per 100,000 between 2008 and 2018. Because decompressive craniectomies were not included, the true incidence of acute surgical procedures following TBI is likely slightly higher.

In conclusion, the incidence of acute craniotomies following TBI has gradually decreased during the last two decades. Simultaneously, the age of neurosurgically treated patients with TBIs has increased. The majority of patients requiring trauma neurosurgery are men and require surgery due an acute SDH. Further studies are needed to determine the indications and derive evidence-based guidelines for the neurosurgical care of older adults with TBIs to meet the challenges of the growing elderly population.

## Data Availability

The original pseudonymized data is not freely available according to the GDPR regulations.

## Code availability

The code and analyses used are available per request.
